# Novel sulfur-doped single-ion conducting multi-block copolymer electrolyte

**DOI:** 10.3389/fchem.2022.974202

**Published:** 2022-08-23

**Authors:** Alexander Mayer, Tugce Ates, Alberto Varzi, Stefano Passerini, Dominic Bresser

**Affiliations:** ^1^ Helmholtz Institute Ulm (HIU), Ulm, Germany; ^2^ Karlsruhe Institute of Technology (KIT), Karlsruhe, Germany

**Keywords:** single-ion conductor, polymer electrolyte, lithium battery, hybrid electrolyte, thiophosphate

## Abstract

Solid-state lithium batteries are considered one of the most promising candidates for future electrochemical energy storage. However, both inorganic solid electrolytes (such as oxide-based or sulfide-based materials) and polymer electrolytes still have to overcome several challenges to replace the currently used liquid organic electrolytes. An increasingly adopted approach to overcome these challenges relies on the combination of different electrolyte systems. Herein, we report the synthesis and characterization of a novel sulfur-doped single-ion conducting multi-block copolymer (SIC-BCE) system. This SIC-BCE may serve as interlayer between the electrodes and the sulfidic electrolyte such as Li_6_PS_5_Cl, thus benefitting of the high ionic conductivity of the latter and the favorable interfacial contact and electrochemical stability of the polymer. The polymer shows excellent ionic conductivity when swollen with ethylene carbonate and allows for stable stripping/plating of lithium, accompanied by a suitable electrochemical stability towards reduction and oxidation. First tests in symmetric Cu|SIC-BCE|Li_6_PS_5_Cl|SIC-BCE|Cu cells confirm the general suitability of the polymer to stabilize the electrode|electrolyte interface by preventing the direct contact of the sulfidic electrolyte with, e.g., metallic copper foils.

## Introduction

Tremendous research efforts are made worldwide to develop and optimize the currently employed and potential future battery technologies (Tarascon and Armand, 2001; Tarascon, 2010; Armand et al., 2020). One major target in this regard is the improvement of the battery safety, which might be achieved, for instance, by replacing the presently used flammable and hazardous liquid organic electrolytes by solid-state ionic conductors (Kalhoff et al., 2015; Janek and Zeier, 2016; Bresser et al., 2018) Generally, there are two major classes of solid electrolyte systems, i.e., inorganic/ceramic materials, such as oxide-based or sulfide-based electrolytes, and polymer-based electrolyte systems (Fan et al., 2018). Each of these classes provides its own benefits and disadvantages. Polymer electrolytes, for example, offer advantageous interfacial contact with the electrodes owing to their flexibility and adhesion properties as well as high electrochemical stability, especially towards lithium metal, while achieving high ionic conductivities remains a challenge (Bresser et al., 2019; Mayer et al., 2021). Differently, sulfide-based electrolytes, for instance, show high ionic conductivity (Minami et al., 2006; Kamaya et al., 2011), but suffer from a relatively narrow electrochemical stability window (Zhu et al., 2015). This renders the direct contact with high-energy and high-voltage cathodes such as Ni-rich LiNi_1–*x*–*y*_Co_*x*_Mn_*y*_O_2_ (NCM; e.g., NCM_622_ or NCM_811_) and lithium metal anodes essentially impossible (Zuo et al., 2021).

To address this challenge, the use of polymeric interlayers, resulting in so-called hybrid electrolyte systems, has been proposed in order to suppress the reductive and oxidative decomposition at the electrode|electrolyte interfaces (Keller et al., 2018; Popovic et al., 2021). Initial attempts focused on the combination of sulfidic electrolytes with poly(ethylene oxide) (PEO) comprising a conducting salt such as lithium bis(trifluoromethanesulfonyl)imide (LiTFSI; Xu et al., 2018; Li et al., 2019). For the hybrid system comprised of PEO-LiTFSI and argyrodite-type Li_6_PS_5_Cl, Simon and co-workers reported a low interfacial resistance and activation barrier for the charge transfer across the PEO-LiTFSI|Li_6_PS_5_Cl interface in symmetric Li||Li cells (Simon et al., 2019). However, the need for elevated temperatures to ensure suitable ionic conductivity of the PEO-LiTFSI system (in this case 80°C) triggered interfacial decomposition reactions, which were attributed to a nucleophilic attack of the ether oxygen in PEO on the phosphorus center of Li_6_PS_5_Cl. Accordingly, stabilizing the ether bonds or completely avoiding their presence in the polymer appears necessary for realizing a sufficiently stable interface. Following these considerations, Li et al. (Li et al., 2020) reported a composite electrolyte composed of Li_3_PS_4_ and *in situ* polymerized poly(ethylene sulfide) (PES), targeting a better compatibility of the two solid electrolyte systems. Nonetheless, also PES–just like PEO–suffers from limited electrochemical stability towards oxidation, rendering it incompatible with NCM-based positive electrodes. Additionally, the use of a conducting salt incorporated in a polymer matrix such as PES or PEO results in rather low lithium-ion transference number (*t*_Li_^+^ <0.3) (Hallinan and Balsara, 2013; Mindemark et al., 2018; Bocharova and Sokolov, 2020). This leads to the potential evolution of ionic concentration gradient limiting the cycle life of the battery cell (Doyle et al., 1994). To generally address these challenges (independent from the combination with an inorganic solid-state electrolyte), Iojoiu, Bresser and co-workers developed a poly(arylene ether sulfone)-based single-ion conducting multi-block copolymer electrolyte (SIC-BCE) with a stabilized ether bond, comprising small ‘molecular transporters’ such as ethylene carbonate (EC) or propylene carbonate (PC). This SIC-BCE provides a high electrochemical stability of >4.5 V and, thus, enables stable cycling of NCM_111_|SIC-BCE|Li cells (Nguyen et al., 2018), NCM_622_|SIC-BCE|Li cells (Steinle et al., 2022), and even NCM_811_|SIC-BCE|Li cells (Chen et al., 2020).

In this work, we followed up on these previous studies and modified the SIC-BCE architecture by doping the polymer with sulfur in order to enhance the compatibility with sulfidic solid-state electrolytes such as Li_6_PS_5_Cl. This modification results in a very high ionic conductivity and stable cycling in symmetric Li‖Li cells, while the oxidative stability is slightly reduced owing to the thioether moiety. Nonetheless, preliminary tests on a layered SIC-BCE|Li_6_PS_5_Cl|SIC-BCE setup suggest that this combination might be suitable to realize hybrid electrolyte systems for high-performance lithium-metal batteries.

## Methods

*Synthesis of the Sulfur-doped Polymer Backbone*: The synthesis of the polymer electrolyte was performed according to previous studies (Assumma et al., 2015a, 2015b; Nguyen et al., 2018) with some minor modifications. In brief, the polymerization of the multi-block copolymer backbone is performed in a one-pot-two-step reaction, followed by bromination and substitution of these reactive sites by the lithium-containing side chains via a copper-catalyzed Ullmann-type coupling reaction. A detailed description of the synthesis and the intermediate products is provided in the Supplementary Material. The synthesis of the lithium-containing sidechain (I-psiLi) was performed following a previously reported synthesis route (Nguyen et al., 2018).

*Membrane Fabrication and Swelling with Ethylene Carbonate:* Polymer membranes were prepared via solvent casting by dissolving 1 g of the polymer electrolyte powder in 10 ml of DMSO (1:10 w/v) on a roll mixer at room temperature. Subsequently, the solution was centrifuged (30 min, 6,000 rpm) to remove any solid impurities and air bubbles. The transparent brownish solution was then poured into a Petri dish (ca. 11 cm in diameter) covered with a perforated aluminum foil to slowly evaporate the solvent at 70°C. The procedure yielded thin polymer films of around 50 µm (±10 µm). The polymer membrane was further dried in vacuo at 150°C for 24 h and immersed in demineralized H_2_O for 48 h to evaporate and dissolve residual traces of solvent. Round discs (18 mm in diameter) were punched out and dried at 130 °C sandwiched between two Petri dishes for 24 h. The resulting membranes were stored in dry room atmosphere (dew point <−65°C). Finally, polymer electrolyte membranes swollen with ethylene carbonate (EC) were fabricated by immersing the polymer membrane in molten EC on a hot plate at 45 °C. An annealing step in a climatic chamber (Binder KB23) at 40 °C for at least 24 h guaranteed equal distribution of the EC in the polymer membrane. The solvent content (*SC*, wt.-%) was calculated using Eq 1 and the weight of the dry (*W*_d_) and the swollen membranes (*W*_s_), and was 55% (±1%), if not stated otherwise:

$$\mathrm{SC}=\frac{W_{s}-W_{d}}{W_{s}}\;\cdot \;100\%$$ (1)

*Physicochemical Characterization:* Structural details of the synthesized polymers and intermediate products were studied by ^1^H and ^19^F nuclear magnetic resonance (NMR) spectroscopy (Bruker Ascend 400 MHz). The molecular weight distribution was determined by gel permeation chromatography (GPC, Malvern Panalytical OmniSEC multi-detector system) using 0.05M LiBr in DMF as the eluent (further details are provided in the Supplementary Material). Differential scanning calorimetry (DSC, Discovery series, TA Instruments) was performed with a heating rate of 5 K min^−1^ (∼10 mg in sealed aluminum pans, -100–230°C, N_2_ gas flow: 10 ml min^−1^). Thermogravimetric analysis (TGA, Netzsch TG 209 F1) was carried out with a heating rate of 5 K min^−1^ in sealed aluminum pans between 30 and 600°C under synthetic air (N_2_/O_2_ 80:20 v/v) using a sample mass of approximately 2 mg.

*Electrochemical Characterization:* Ionic conductivity measurements were conducted in symmetric Cu||Cu cells at different temperatures. The polymer electrolyte membranes were sandwiched between two battery-grade copper foils in pouch cells in a dry room. After sealing the pouch cells using a vacuum sealer (Audiovac VMS 163, Audion), the cells were stored at 40 °C for at least 24 h prior to the measurements to allow for a homogenization of the interface. Electrochemical impedance spectroscopy (EIS) was performed using a Solartron SI 1260/1287 Impedance Analyser (frequency range: 1 Hz to 1 MHz) at different temperatures in a Binder climatic chamber KB23 with 3 h rest after decreasing or increasing the temperature prior to the next measurement. The subsequent analysis of the data was carried out with the RelaxIS 3 software (rhd instruments), applying an *RP* fitting model. The ionic conductivity (*σ*) was determined *via* Eq 2, taking into account the thickness *d* of the polymer membrane (after the measurement, determined with a Mitutoyo Absolute digital thickness gauge 547-401) and the area *A* of the polymer membrane covered by both copper electrodes:

$$\sigma =\frac{d}{\mathrm{RA}}\;$$ (2)

Lithium stripping/plating experiments were conducted in coin cells (CR 2032, Hohsen) at 40°C (Binder climatic chamber KB 115), using a Maccor 4000 battery testing system. Disk-shaped lithium foils (14 mm in diameter, 500 µm thickness, battery grade, Honjo) were placed on spacers made of stainless steel (16 mm, 0.5 mm thickness), followed by sandwiching the EC-doped polymer membranes between the lithium disks in an argon-filled glovebox (MBRAUN MB-200-MOD, H_2_O/O_2_ <1 ppm). The coin cells were sealed using a hydraulic coin cell crimping machine (MSK-110, MTI Corp, pressure of ∼800 psi). The current density was gradually varied from 5 to 10, 20, 50, 100, 200 and 500 μA cm^−2^, and the current was reversed after 1 h intermitted by a 5 min rest step.

The measurement of the Li^+^ transference number $t_{{\text{Li}}^{+}}$ was performed following the ‘polarization method’ proposed by Bruce, Vincent and Evans in 1987 (Evans et al., 1987). Therefore, a symmetric Li||Li coin cell was assembled as described above and stored at 40 °C in a climatic chamber (Binder) for 48 h to allow for thermal equilibration. The cell was polarized with 10 mV and the current was recorded until a steady state was reached. For this experiment, a Solartron SI 1260/1287 Impedance Analyzer (frequency range: 1 Hz to 1 MHz) was used. Impedance measurements were conducted before and after the polarization of the cell and analyzed using the RelaxIS 3 software (rhd instruments). Following the suggestions in the literature (Zhao et al., 2008; Zugmann et al., 2011), $t_{{\text{Li}}^{+}}$ was finally calculated by using Eq 3, where $I_{0}$ and $I_{\text{ss}}$ represent the current measured right after the polarization and when the steady state was reached. $R_{0}$ and $R_{\text{ss}}$ refer to the resistance before polarization and at steady state and were measured by electrochemical impedance spectroscopy (EIS). ΔV describes the polarization applied to the cell.

$$t_{Li^{+}}=\frac{I_{\mathrm{ss}}\left(\Delta V-I_{0}R_{0}\right)}{I_{0}\left(\Delta V-I_{\mathrm{ss}}R_{\mathrm{ss}}\right)}$$ (3)

The electrochemical stability window was determined via linear sweep voltammetry (LSV). For this purpose, two-electrode pouch cells with nickel foil as the working electrode and lithium foil (50 µm thickness, battery grade, Honjo) as the counter electrode were assembled in a dry room. The measurements were performed with a BioLogic VMP3 Multichannel Potentiostat at 40°C (Binder climatic chamber KB23) and a sweep rate of 1 mV s^−1^ after a rest time of 24 h. The cut-off voltages were set to -2.0 V and +6.0 V. Freshly assembled cells were used for each LSV experiment.

To investigate the general compatibility of the polymer electrolyte with the thiophosphate electrolyte Li_6_PS_5_Cl (Ampcera™, MSE Supplies, pellets pressed at 360 MPa for 30 s, 12.8 mm diameter), conductivity measurements were performed in Torque cells applying a pressure of 5 Nm with a torque wrench. The cell setup, measurement, and data evaluation followed the conductivity measurements on the bare polymer electrolyte, as described above. The pressed Li_6_PS_5_Cl pellet (750 µm thickness) was sandwiched between two SIC-BCE membranes (12 mm diameter, ca 120 µm thickness) on copper foil. The overall thickness of the setup was estimated to be about 1 mm.

## Results and discussion

**FIGURE 1:**
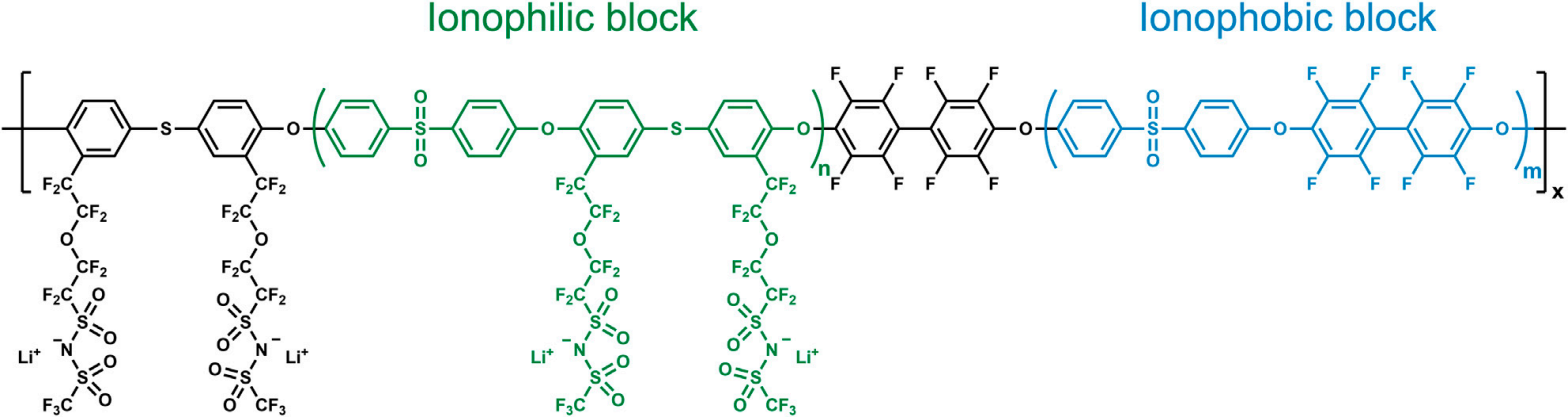
Chemical structure of the sulfur-doped SIC-BCE.

The chemical structure of the single-ion conducting polymer is presented in Figure 1. Structures of the precursor polymers can be found in the synthesis scheme provided in Supplementary Figure S1. While the ^1^H NMR spectrum of the first (later the ionophilic) block (sample extracted right before the addition of the monomers of the second block) showed the expected signals and integrals, a minor signal of unreacted decafluorobiphenyl (DFBP) monomers was detected in the ^19^F NMR spectrum (Supplementary Figure S2). Subsequently, the ratio of the ionophilic block and the ionophobic block was adjusted to the desired value of 2:1 by adding the corresponding amount of the monomers forming the ionophobic block. The ratio was confirmed by integrating the ^1^H NMR spectrum of the polymer backbone before chemical modification (Supplementary Figure S3). As apparent from the ^19^F NMR spectrum, the decafluorobiphenyl monomers had been completely consumed now. The bromination of the polymer backbone, however, does not lead to a perfectly regioselective dibromination, as reported for the previous SIC-BCE (Nguyen et al., 2018), and the ^1^H NMR spectrum reveals the presence of side products (Supplementary Figure S4), which renders the peak integration and clear allocation challenging. One possibility could be the formation of mono- and/or tribrominated species. Nevertheless, in that case smaller and more distinct peaks would be expected. Another possible explanation is indicated by the comparison of the GPC results obtained for the non-brominated and the brominated polymer backbone (Supplementary Table S1). Theoretically, one would expect an increased molecular weight for the brominated polymer due to the relatively higher atomic mass of the bromide substituent. However, the molecular weight has decreased after the bromination. This might indicate that the bromination leads to a partial cleavage of the thioether bonds, which are suggested to be less stable compared to oxygen ether bonds, and the formation of relatively shorter molecules. The overall effect of such potential partial bond cleavage, though, remains limited, since the subsequent attachment of the lithium-bearing side chain via an Ullmann-type coupling reaction was successful, as proven by ^19^F NMR spectroscopy (Supplementary Figure S5). The spectrum shows signals of the ionophobic block and signals of the side chain tethered to the ionophilic block, which was also confirmed by the increase in molecular weight (Supplementary Table S1). Following the successful synthesis (despite potentially shortened polymer chains) and the realization of self-standing membranes, also after incorporating ethylene carbonate (EC) as charge-transport supporting small molecules, the resulting polymer-based electrolyte system was subjected to a comprehensive physicochemical and electrochemical characterization.

**FIGURE 2:**
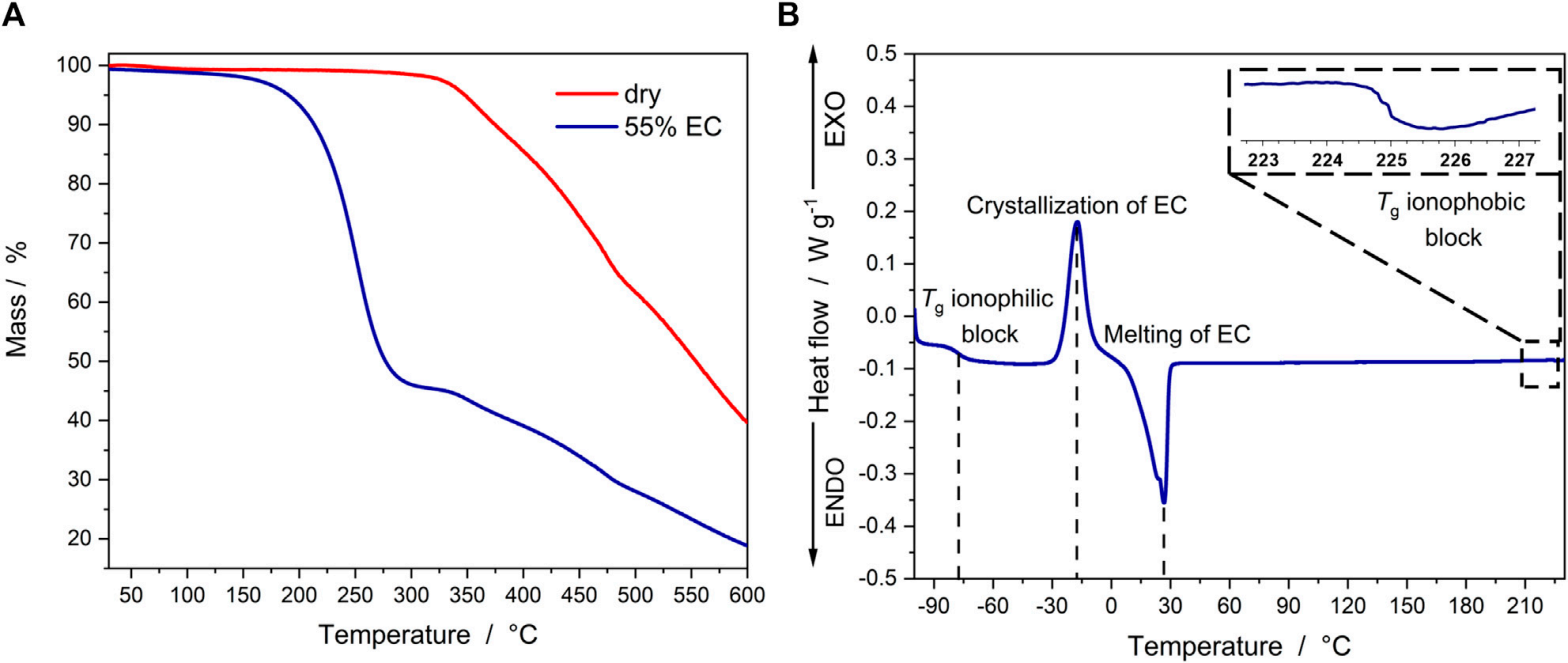
**(A)** TGA data recorded for the sulfur-doped SIC-BCE without EC (red) and swollen with 55% EC (blue). **(B)** DSC curve of the SIC-BCE membrane swollen with 55% EC.

The characterization via TGA revealed a thermal stability of more than 300 °C for the as-synthesized ionomer (Figure 2A), which is in line with the previous results for the ether-type SIC-BCE (Nguyen et al., 2018). The TGA measurement of a membrane swollen with 55% EC reveals a stable behavior up to 150°C, followed by the evaporation of EC (completed around 300°C, as indicated by the mass loss of ca. 55%) and the regular decomposition of the polymer starting approximately at 300 °C. Further characterization of the (55 -wt%) EC-doped membranes via DSC (Figure 2B) showed that the glass transition temperature (*T*_g_) of the ionophilic block is around -77°C. This is significantly lower than the value of -40°C reported for the polymer electrolyte without thioether moieties (Nguyen et al., 2018) and promises a higher ionic conductivity for the sulfur-doped SIC-BCE. The *T*_g_ of the ionophobic block was found to be around 225°C, which is essentially the same as for the non-sulfur-doped SIC-BCE reported earlier by Nguyen et al., which indicates that also in the case of the thioether-comprising polymer the EC molecules are preferentially coordinating the ionophilic domains. The additional presence of free EC, i.e., EC molecules that are not directly coordinating the ionophilic domains, is reflected by an exothermic peak at about -17°C that is assigned to the cold crystallization of this free EC, followed by an endothermic melting peak at about 30 °C.

**FIGURE 3:**
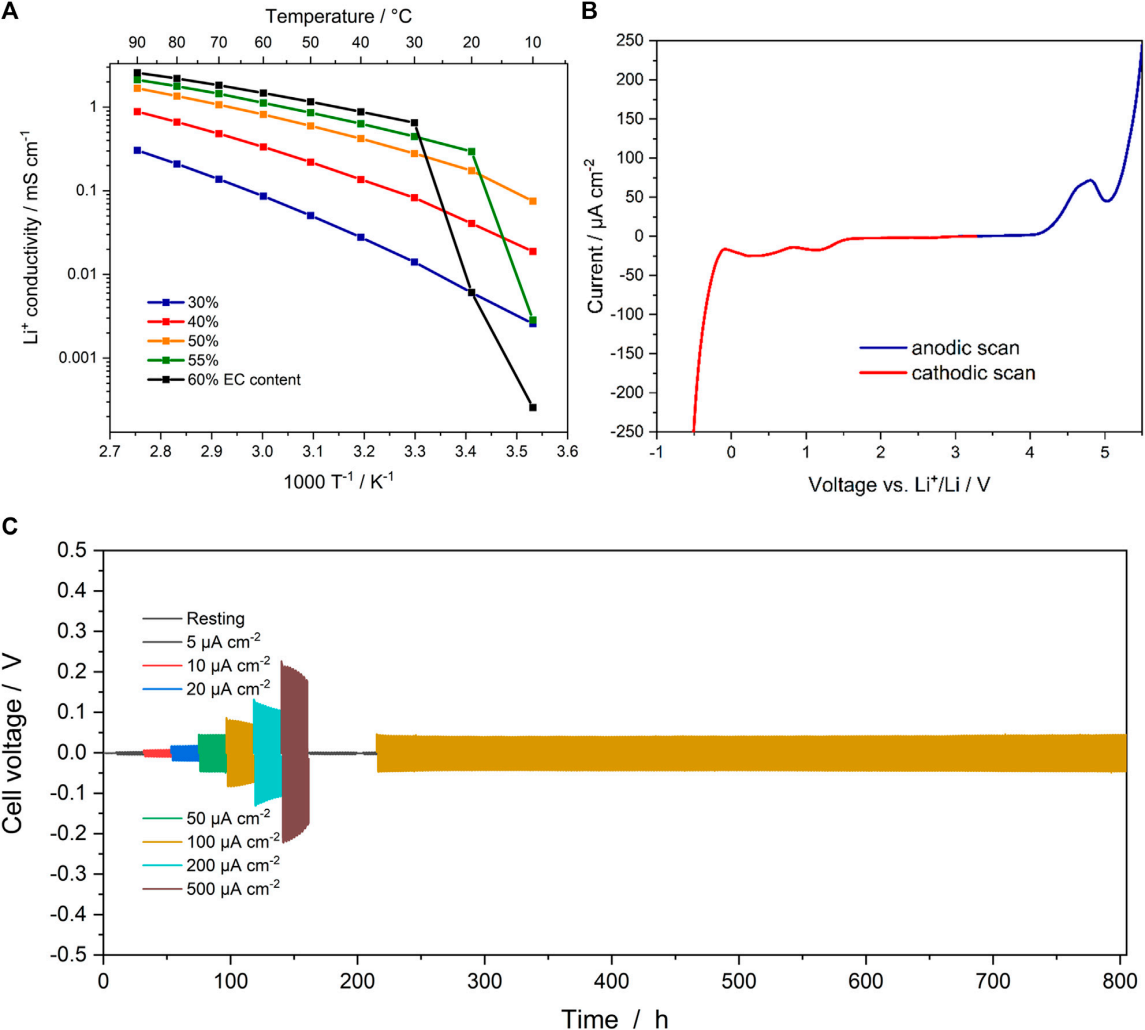
**(A)** Ionic conductivity of the sulfur-doped SIC-BCE with 30, 40, 50, 55 and 60% EC content between 10 and 90°C. **(B)** Electrochemical stability window of the sulfur-doped SIC-BCE with 55% EC at 40°C. **(C)** Overpotential at varying current densities during lithium stripping/plating at 40°C, followed by stripping/plating at 5 and 100 μA cm^−2^ at 40°C (including a rest step).

Subsequently, the ionic conductivity of the sulfur-doped SIC-BCE was determined via EIS between 10 and 90 °C for various EC contents (i.e., 30, 40, 50, 55 and 60%). The results are presented in Figure 3A. Generally, the ionic conductivity increases for an increasing EC content and reaches more than 1 mS cm^−1^ for an EC content of ≥50% at elevated temperatures. At 40 °C, the conductivity of the ionomer comprising 55% EC is still as high as 0.6 mS cm^−1^. Below 20°C, though, it rapidly drops owing to the crystallization of the free EC domains, which are presumably blocking the conductive channels in the polymer-based electrolyte. In fact, for an even higher EC content, this effect is even more dramatic, while it does not occur for EC contents lower than 50%. The corresponding Nyquist plots obtained at 10 °C are presented in Supplementary Figure S6. The correlation to the presence of free EC domains, i.e., EC that does not strongly coordinate the ionophilic domains, above the threshold of about 50% is also well reflected by the DSC traces for the different polymer-based electrolyte systems, as displayed in Supplementary Figure S7. The SIC-BCE systems comprising 30 and 40% EC do not show any EC melting-related endothermic peak, since all EC is strongly coordinated to the ionophilic domains of the ionomer. For 50% EC, a very tiny endothermic peak is observed, which is in line with the minor decrease in conductivity at 10 °C, slightly deviating from the general trend. Beyond 50% a pronounced melting peak is observed, corroborating the presence of non-coordinating, i.e., free EC domains. Interestingly, the ionomer comprising 60 and 65% EC do not show any indication of a cold crystallization of EC, but rather a crystallization during the cooling cycle (not shown herein). This might be related to the amount of free EC, which is significantly larger in these two cases, favoring a kinetically controlled crystallization already upon cooling.

The electrochemical stability was determined *via* LSV (Figure 3B). For the cathodic sweep, two broad peaks of low intensity are observed at 1.15 and 0.35 V, which are assigned to traces of DMSO (Yamada et al., 2010), remaining from the membrane casting process, and the reductive decomposition of EC (Zhang et al., 2001), respectively. Below 0 V, lithium plating occurs, as indicated by the extensive current evolution. During the anodic sweep, prior to the rapid increase in current at about 5 V, a broad peak with a maximum intensity at about 4.8 V is observed. In part, this peak has been assigned to the oxidative decomposition of the DMSO traces and EC (Nguyen et al., 2018). The onset of this peak, however, occurs at about 4.2 V, i.e., substantially lower than for the SIC-BCE without the thioether moiety (Nguyen et al., 2018; Chen et al., 2020), indicating that the presence of the thioether function leads to a slight reduction in electrochemical stability towards oxidation. Nonetheless, this limitation might be overcome by carefully oxidizing the thioether group to S=O or O=S=O moieties; potentially, though, at the expense of a slightly reduced ionic conductivity (Sarapas and Tew, 2016).

To confirm the compatibility with lithium-metal electrodes, lithium stripping/plating experiments were conducted for symmetric Li||Li cells (Figure 3C). The current density was stepwise increased from 5 μA cm^−2^ to 500 μA cm^−2^ and then decreased back to 5 μA cm^−2^ and, eventually, 100 μA cm^−2^. In general, the overpotential remained rather low for all current densities with max. around 0.2 V at 500 μA cm^−2^, and no sign of dendrite formation/short circuits was observed. The overpotential at 5 μA cm^−2^ was essentially the same before and after applying elevated current densities, highlighting the good compatibility with metallic lithium. For the subsequent stripping/plating at 100 μA cm^−2^ for more than 500 h, a stable and slightly lower overpotential was observed compared to the overpotential recorded at the same current density before subjecting the cell to 200 and 500 μA cm^−2^. This is attributed to an increased surface area of the lithium-metal electrodes, i.e., a relatively lower current density per surface area, as also indicated by the slowly decreasing overpotential at elevated current densities, when significantly more lithium is stripped and plated during each cycle. In either case, however, the voltage response reveals an essentially perfectly rectangular shape, as shown in Supplementary Figure S8, which provides a zoom into the stripping/plating experiment at 100 μA cm^−2^ after about 300 h. This constant voltage response indicates the expected single-ion conducting behavior, as also further corroborated by the determination of the Li^+^ transference number according to Eq 3 (see also Supplementary Figure S9), yielding a value of $t_{{\text{Li}}^{+}}$ ≈ 0.97, i.e., essentially unity. The very small difference might be related to the presence of very minor traces of smaller molecules (e.g., short oligomers) that are initially polarized even despite a macroscopic charge neutrality (Liang et al., 2022).

**FIGURE 4:**
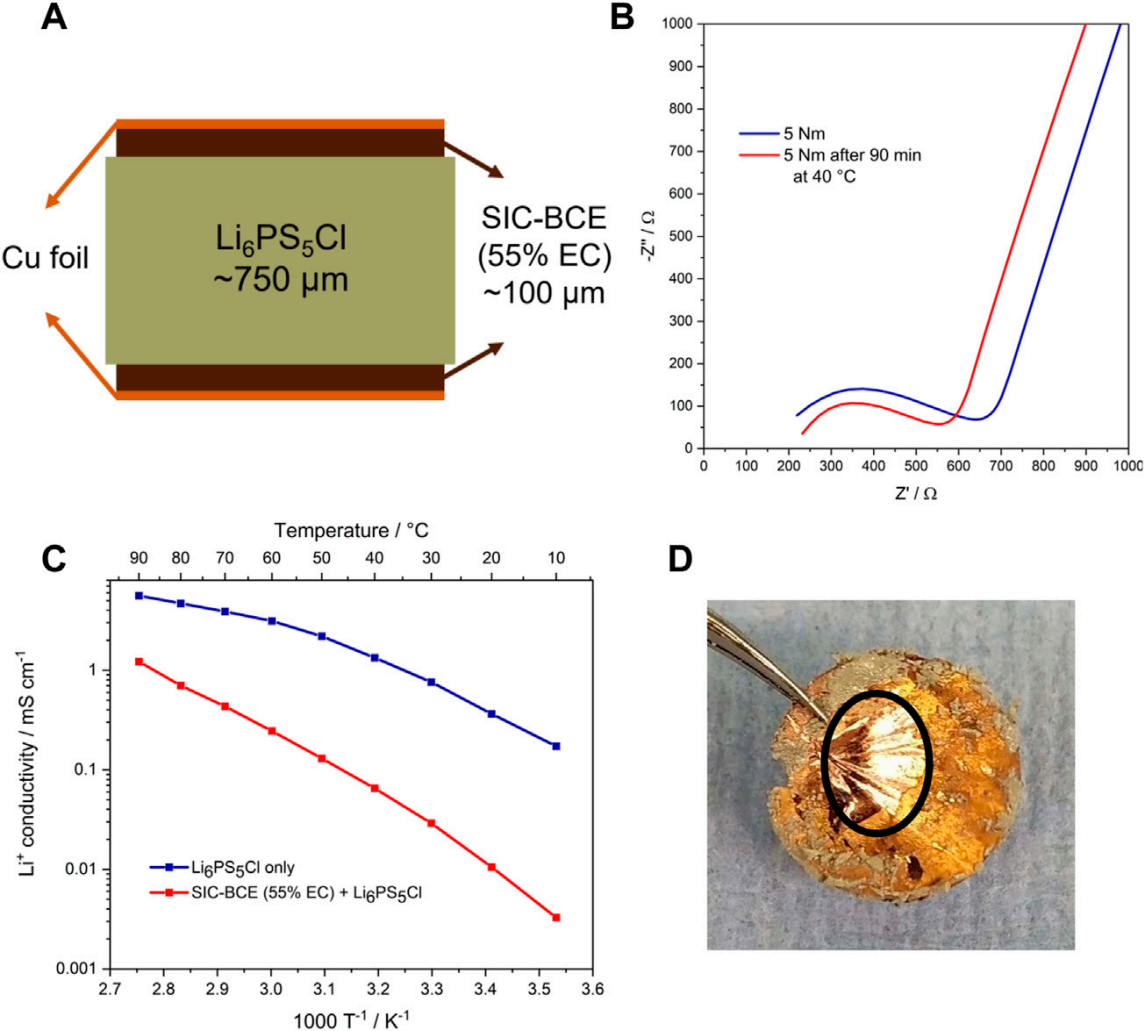
**(A)** Scheme of the cell setup used for the conductivity measurements of the SIC-BCE as interlayer between the copper current collector and solid-state thiophosphate electrolyte. **(B)** Nyquist plots of the impedance data obtained right after applying pressure to the cell and after 90** **min storage at 40°C. **(C)** Comparison of the ionic conductivity of pure thiophosphate pellets (blue) and the layered hybrid system with the thiophosphate pellet sandwiched between to SIC-BCE membranes on copper foil (red). **(D)** Photograph of the copper foil after the conductivity tests with some residual thiophosphate (greenish residues). The part outside the black circle was still covered by the SIC-BCE membranes, while it had been removed for the part within the black circle, revealing a shiny copper surface.

Finally, to investigate the general compatibility of this new sulfur-doped SIC-BCE with a thiophosphate-based electrolyte, i.e., Li_6_PS_5_Cl, symmetric Cu|SIC-BCE|Li_6_PS_5_Cl|SIC-BCE|Cu cells were assembled as depicted in Figure 4A. These cells were subjected to EIS measurements in order to determine the ionic conductivity. To start with, EIS spectra were recorded right after cell assembly and applying a pressure of 5 Nm as well as after 90 min storage at 40°C (Figure 4B). The impedance decreased upon storage at such slightly elevated temperature, indicating that the system adjusted the applied pressure, presumably *via* diffusion of the polymer and the comprised EC. This process, though, was completed after 90 min, as no further changes were observed for longer storage times (not shown herein). The ionic conductivity of such 2D hybrid electrolyte reached more than 1 mS cm^−1^ at 90 °C and more than 0.1 mS cm^−1^ at 50°C (Figure 4C). While these values are a little lower than for the neat SIC-BCE and also lower than those recorded for the neat Li_6_PS_5_Cl, it is anticipated that it can be well increased by optimizing the charge transfer at the SIC-BCE|Li_6_PS_5_Cl interface in future studies. More important for this proof-of-concept experiment was the subsequent disassembly of the cell and the inspection of the copper foil. In fact, Li_6_PS_5_Cl is generally incompatible with metallic copper foil (just like with metallic lithium (Wenzel et al., 2018)), and the direct contact results in the formation of ionically and electronically conducting Cu_x_S (Homann et al., 2020) and, consequently, the degradation of the copper foil and the sulfidic electrolyte. The photograph depicted in Figure 4D shows that some Li_6_PS_5_Cl sticked to the SIC-BCE-coated copper foil, while there was no indication of copper foil degradation observed. In fact, when removing part of the SIC-BCE from the copper foil, a very shiny and corrosion-free metallic surface was found (see the black circle in Figure 4D), confirming the general objective of introducing a protective SIC-BCE interlayer that physically prevents any direct contact between the electrode and the sulfidic electrolyte, while allowing for a suitable charge transfer across this interlayer and the additional interface.

In conclusion, we presented the successful synthesis of a new single-ion conducting multi-block copolymer electrolyte, comprising thioether groups to enhance the compatibility with sulfidic solid-state electrolytes when used in hybrid electrolyte systems. This new polymer electrolyte provides high ionic conductivity of more than 1 mS cm^−1^ at elevated temperatures and about 0.6 mS cm^−1^ at 40°C when incorporating 55% EC. The electrochemical stability towards oxidation is slightly reduced as a result of the thioether moiety, which might be addressed by carefully oxidizing it in future studies. Preliminary tests in a 2D hybrid configuration with Li_6_PS_5_Cl confirm the general suitability of this new polymer for such combination and the successful protection of the copper current collector. Future studies will be dedicated to a more detailed investigation and optimization of such hybrid systems and the long-term stability of the relevant interfaces (and potentially formed interphases).

## Data Availability

The original contributions presented in the study are included in the article/Supplementary Material; further inquiries can be directed to the corresponding authors.
