# Supplementary material for: Novel sulfur-doped single-ion conducting multi-block copolymer electrolyte
Source: Front Chem. 2022 Aug 23;10:974202. doi: 10.3389/fchem.2022.974202 (PMC9445549; doi:10.3389/fchem.2022.974202)
Supplement: Supplementary file 1 [file DataSheet1.docx]

**Supplementary Material**

**Experimental Section**

**1. Materials for Synthesis**

*N*,*N*-Dimethylacetamide (DMAc, anhydrous, 99.5%), triethylamine (TEA, 99.7%), aluminum oxide (neutral, Brockmann I, 40-300 µm, 60 Å), bromine (99.6%) and 4,4’-difluorodiphenyl sulfone (DFDPS, >99%) were purchased from Acros Organics. VWR supplied dimethylacetamide (DMAc, GPR Rectapur), dimethyl sulfoxide (DMSO, GPR Rectapur), dichloromethane (DCM, GPR Rectapur, stabilized with 0.002% 2-methyl-2-butene), ethyl acetate (EtOAc, GPR Rectapur), methanol (MeOH, GPR Rectapur), anhydrous magnesium sulfate (MgSO_4_, 99.5%), hydrochloric acid (37%) and acetonitrile (99.8%, anhydrous). Dimethyl sulfoxide (DMSO, anhydrous, 99.8%), toluene (anhydrous, 99.8%), 4,4’-thiodiphenol (BP-Thio, 98%+), anhydrous lithium hydroxide (LiOH, 98%), copper (Cu) powder (99%, average particle size ~100 mesh/~150 μm), and 4,4’-dihydroxydiphenyl sulfone (DHDPS, 99%) were purchased from Alfa Aesar. Potassium carbonate (K_2_CO_3_, 99%) and acetic acid (AcOH, 99-100%) were supplied by Merck, anhydrous ethylene carbonate (EC, 99%) by Sigma-Aldrich. abcr supplied decafluorobiphenyl (DFBP, 99%). 1,1,2,2-tetrafluoro-2-(1,1,2,2-tetrafluoro-2-iodoethoxy)ethane sulfonyl fluoride (ICF_2_CF_2_OCF_2_CF_2_SO_2_F, 95%) and trifluoromethanesulfonamide (CF_3_SO_2_NH_2_, 98%) were purchased from Matrix Scientific. All chemicals were used as received. *N*,*N*‑Dimethylformamide (DMF, ≥99.8%) and anhydrous lithium bromide (LiBr, 99+%) for the GPC experiments were purchased from Acros Organics. The solvents used for the NMR spectroscopy experiments were purchased from Acros Organics (CDCl_3_, 99.8+% D + 0.03% (v/v) TMS) and VWR (acetone-d_6_, 99.8% D and DMSO-d_6_, 99.8% D + 0.03% (v/v) TMS).

**
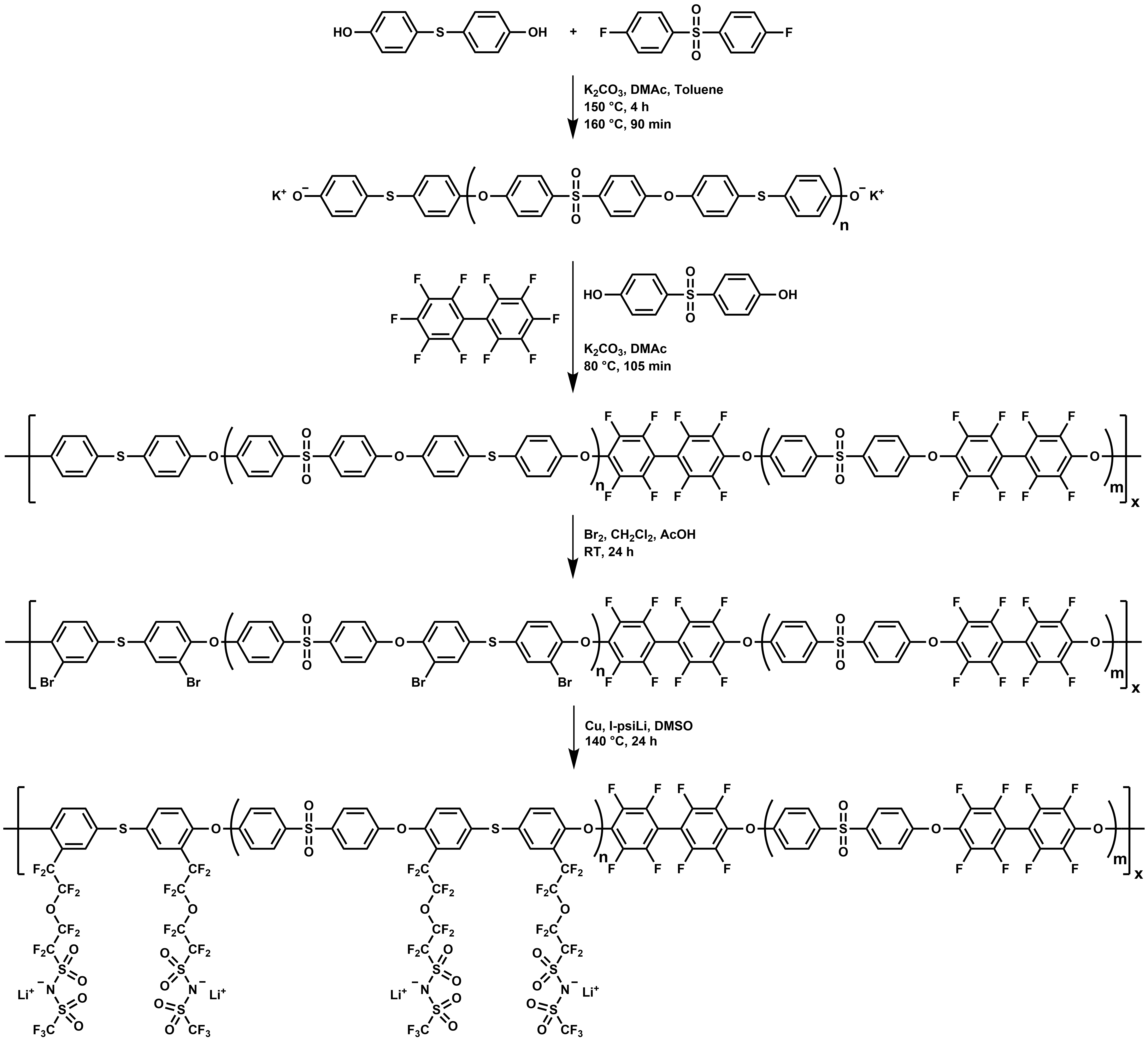
**

**Figure S1:** Synthesis scheme of the sulfur-doped single-ion conducting multi-block copolymer electrolyte.

**2. Polymerization of the sulfur-doped polymer backbone**

4,4’-Thiodiphenol (BP-Thio, 31.792 mmol, 6.939 g, 1 equiv.), 4,4’-difluorodiphenyl sulfone (DFDPS, 31.466 mmol, 8.000 g, 0.99 equiv.), and K_2_CO_3_ (95.262 mmol, 13.166 g, 3 equiv.) were introduced into a pre-dried 250 mL three-neck round bottom flask equipped with a gas inlet, a mechanical stirrer and a Dean-Stark trap, including a condenser with a gas outlet. After evacuating and flushing with argon, 100 mL of dimethylacetamide (DMAc) and 50 mL of toluene (2:1 v/v) were added to dissolve the monomers. Subsequently, the oil bath temperature was increased to 150 °C for 4 h and water was continuously removed by forming an azeotrope with toluene. After 4 h, the temperature was raised to 160 °C and the toluene was removed entirely within 90 min. Then, the already slightly viscous solution was allowed to cool down to 80 °C and an aliquot (<1 mL) was extracted to determine the molecular weight of the first block. After adding K_2_CO_3_ (46.866 mmol, 6.477 g, 1.48 equiv.), a solution of 4,4’‑dihydroxydiphenyl sulfone (DHDPS, 15.622 mmol, 3.910 g, 0.49 equiv.) and decafluorobiphenyl (DFBP, 16.527 mmol, 5.522 g, 0.52 equiv.) in 40 mL DMAc was added to the reaction mixture under argon in order to adjust a 2:1 ratio of the polymer blocks. The reaction was allowed to proceed at this temperature until the viscosity increased considerably (105 min) and cooled down to room temperature afterwards. The viscous reaction mixture was then carefully poured into 2 L of 1M HCl, yielding white filaments, and was stirred overnight. Filtration, followed by washing the polymer with demineralized H_2_O until a neutral pH value was reached and drying at 100 °C in vacuo for 24 h, resulted in 21.6 g of white polymer filaments. To further purify the polymer and receive a more homogeneous material, the polymer filaments were dissolved in 330 mL of DMAc (ca. 1:15 w/v) and the solution was filtered through neutral alumina on a ceramic suction filter (porosity 4). Solid impurities were removed by centrifugation (6000 rpm for 30 min) and the resulting clear solution was precipitated by dropwise adding it to a vigorously stirred solution of 2 L of 1M HCl. After stirring overnight, the white polymer flakes were filtered, washed until a neutral pH was reached and finally dried in vacuo at 100 °C, yielding around 20 g of polymer (yield: ~90%).

**
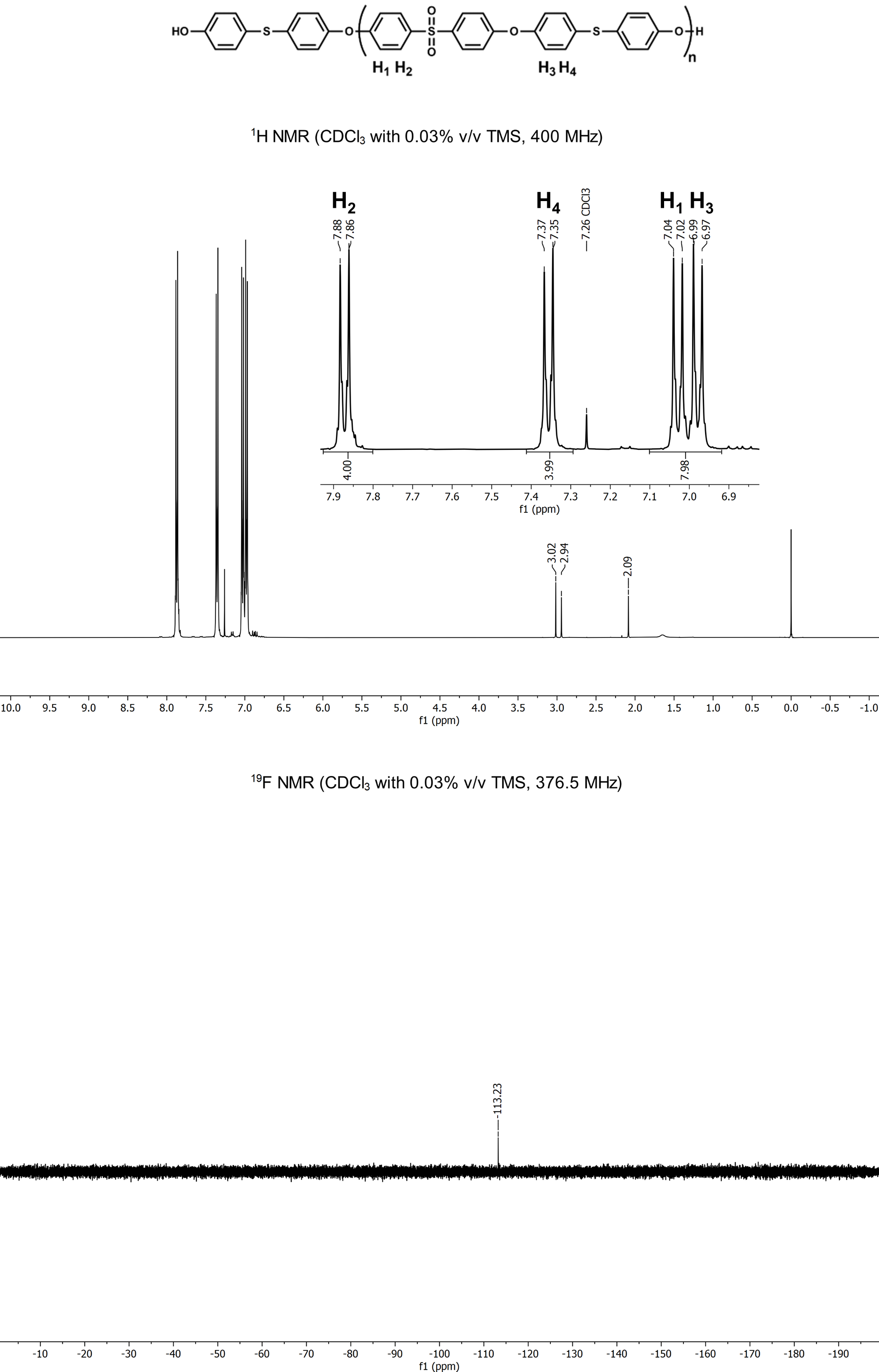
Figure S2**: ^1^H (top) and ^19^F (bottom) NMR spectrum of the 1^st^ (later ionophilic) block of the multi-block copolymer in CDCl_3_. The sample was extracted right before the addition of the monomers forming the 2^nd^ (ionophobic) block.

**
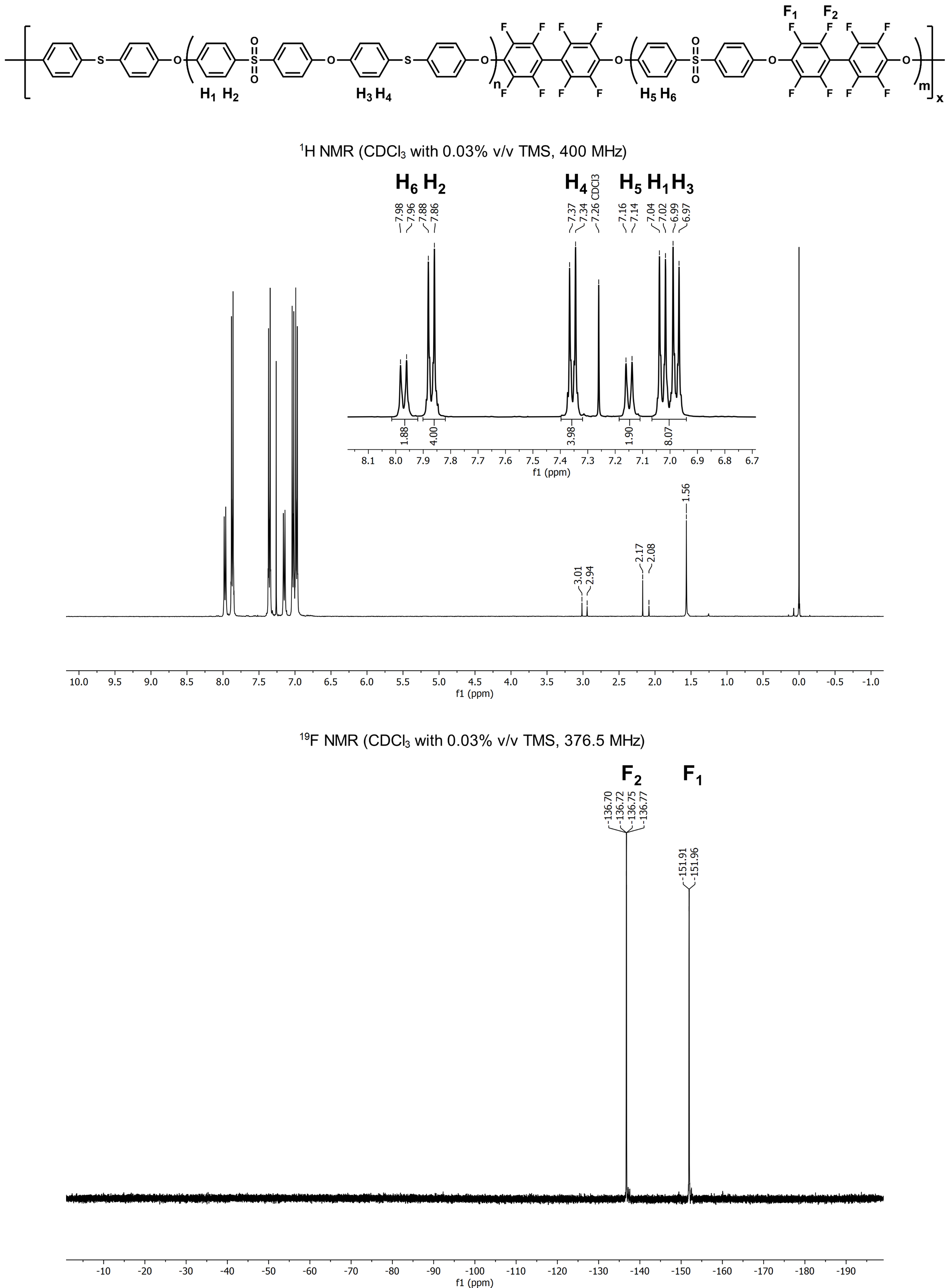
Figure S3**: ^1^H (top) and ^19^F (bottom) NMR spectrum of the multi-block copolymer in CDCl_3_.

**3. Bromination of the sulfur-doped polymer backbone**

10 g (7.095 mmol, 1 equiv., 4 equiv. of reactive sites due to the 2:1 ratio of 1^st^ (ionophilic) and 2^nd^ (ionophobic) block) of the polymer were introduced into a 500 mL pre-dried three-neck round bottom flask equipped with an addition funnel and a condenser. Dichloromethane (DCM, 200 mL) and acetic acid (10:1 v/v, 20 mL) were added to dissolve the polymer. As soon as a clear solution was obtained, bromine (10.9 mL, 212.858 mmol, 30 equiv. in total, 7.5 equiv. per reactive site) was added dropwise and the reaction mixture was stirred for 24 h at room temperature. The resulting red solution was then precipitated into 1 L of methanol. After filtration and repeated washing with methanol and drying in vacuo (80 °C), ~12 g of a fine white powder was obtained. For purification purposes, the polymer was dissolved once more in DCM (150 mL, ca. 1:13 w/v) and filtered through neutral alumina on a ceramic suction filter (porosity 4). Solid impurities were removed by centrifugation (6000 rpm for 30 min) and the polymer in the resulting clear solution was precipitated by dropwise adding it to vigorously stirred methanol. After stirring overnight, the white polymer flakes were filtered, washed with methanol and finally dried in vacuo (80 °C), yielding 10.4 g of brominated polymer (yield: ~85%).

**
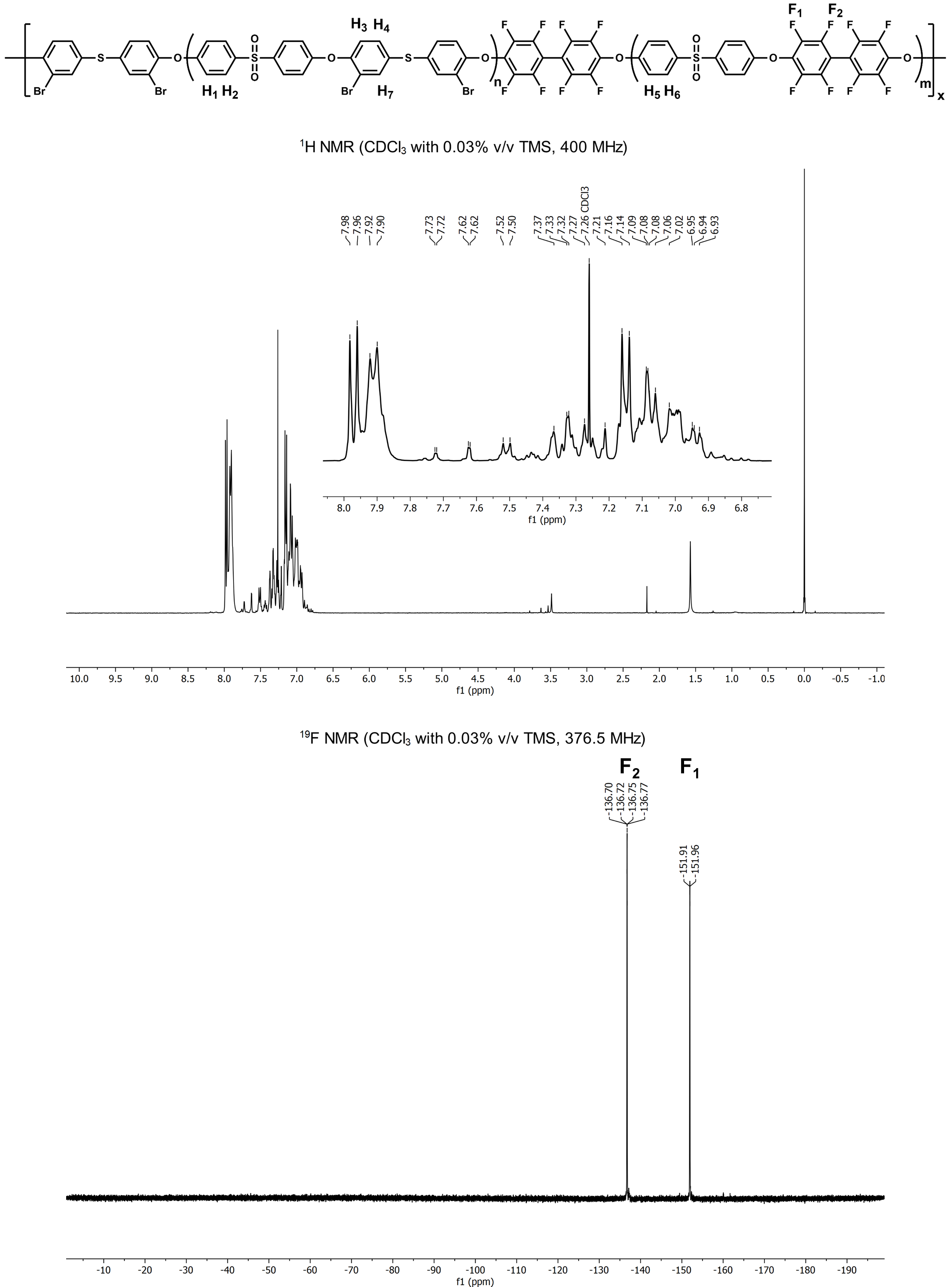
Figure S4**: ^1^H (top) and ^19^F (bottom) NMR spectrum of the brominated multi-block copolymer in CDCl_3_.

**4. Addition of the side chain to the sulfur-doped polymer backbone via copper-catalyzed Ullmann coupling reaction**

5 g (2.899 mmol, 1 equiv., 4 equiv. of brominated reactive sites) of the brominated polymer and 4.421 g (69.566 mmol, 24 equiv. in total, 6 equiv. per brominated reactive site) of copper powder were introduced to a 250 mL pre-dried three-neck round bottom flask, equipped with an addition funnel, a mechanical stirrer and a condenser with a gas inlet. After evacuating and flushing with argon, 50 mL of dimethyl sulfoxide (DMSO) were added and the mixture was heated to 80 °C to dissolve the polymer. Subsequently, the temperature was increased to 120 °C and held constant for 2 h. The I-psiLi component (9.757 g, 17.392 mmol, 6 equiv. in total, 1.5 equiv. per brominated reactive site), dissolved in 33 mL DMSO under argon, was added dropwise to the reaction mixture while the temperature was simultaneously increased to 140 °C. After 24 h, the reaction mixture was allowed to cool down and the copper powder was removed by repeated steps of centrifugation (5 x 1 h at 6000 rpm). The resulting transparent brown solution was then poured into 1 L of 1M HCl. The precipitated polymer was stirred overnight and washed with demineralized H_2_O until a neutral pH was reached. Remaining traces of acid were removed by immersing the polymer particles in 1M LiOH for 15 min, followed by filtration, washing with demineralized H_2_O, and drying in vacuo (80 °C). For purification purposes, 5.9 g of the polymer was dissolved in 30 mL of DMSO (ca. 1:5 w/v), resulting in a viscous brown solution, and was subsequently filtered through neutral alumina on a ceramic suction filter (porosity 4). Solid impurities were removed by centrifugation (6000 rpm for 30 min) and the polymer in the resulting clear solution was precipitated by dropwise adding it to a vigorously stirred solution of 1M HCl. After 2 h, the polymer was filtered, washed and immersed in 1M LiOH under vigorous stirring overnight. Finally, the polymer was filtered and washed again until a neutral pH was reached, followed by a drying step at 80 °C in vacuo and grinding of the polymer particles, resulting in 4.5 g of brown polymer powder (yield: ~70%).

**
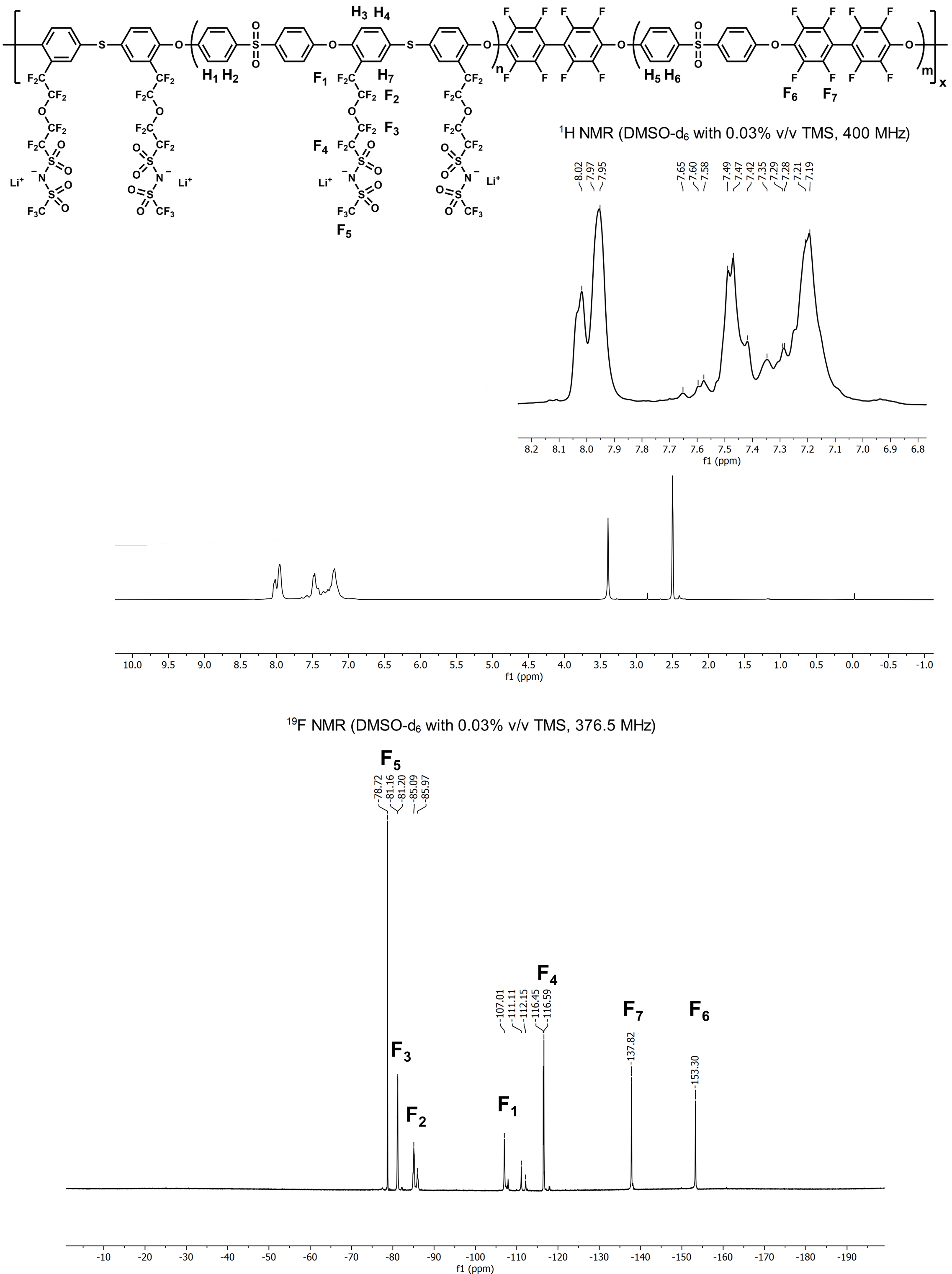
Figure S5:** ^1^H (top) and ^19^F (bottom) NMR spectrum of the single-ion conducting multi-block copolymer in (CD_3_)_2_SO.

**Table S1:** Molecular weights of all synthesized polymers determined by GPC.

|  | ***M*_n_ /**  g mol^-1^ | ***M*_w_ /**  g mol^-1^ | **PDI** |
| --- | --- | --- | --- |
| **1^st^ (later ionophilic) block** | 39,500 | 68,000 | 1.72 |
| **Multiblock copolymer** | 83,000 | 300,000 | 3.61 |
| **Brominated polymer** | 36,500 | 67,500 | 1.85 |
| **Single-ion conducting polymer** | 145,000 | 305,000 | 2.10 |

**5. Size Exclusion/Gel Permeation Chromatography (SEC/GPC)**

The molecular weight of the polymer samples was determined by gel permeation chromatography (GPC) using a multi-detector Malvern Panalytical OmniSEC Resolve/Reveal system equipped with a three-column setup (Viscotek D5000-D3000-D2000). The eluent was a 0.05M LiBr solution in *N,N*-dimethylformamide (DMF) with a flux rate of 0.8 mL min^-1^ at 50 °C. The sample analysis was performed using a refractive index (RI) detector, accompanied by a UV detector, detectors for low-angle and right-angle light scattering (LALS and RALS), as well as a viscometer. The molecular weight was derived from the refractive index and light scattering, utilizing a universal calibration method with one narrow poly(methyl methacrylate) (PMMA) standard, which was subsequently verified by analyzing an additional broad PMMA standard. To prepare the GPC samples, 20-25 mg (weighed precisely) of each polymer sample were dissolved in 10 mL of the eluent, followed by filtration through a syringe filter. Two injections per sample with an injection volume of 100 µL each were carried out to ensure data consistency. The OmniSEC software was used to analyze the data obtained.

**6. Additional Electrochemical and Thermal Characterizations of the Sulfur-Doped SIC-BCE**


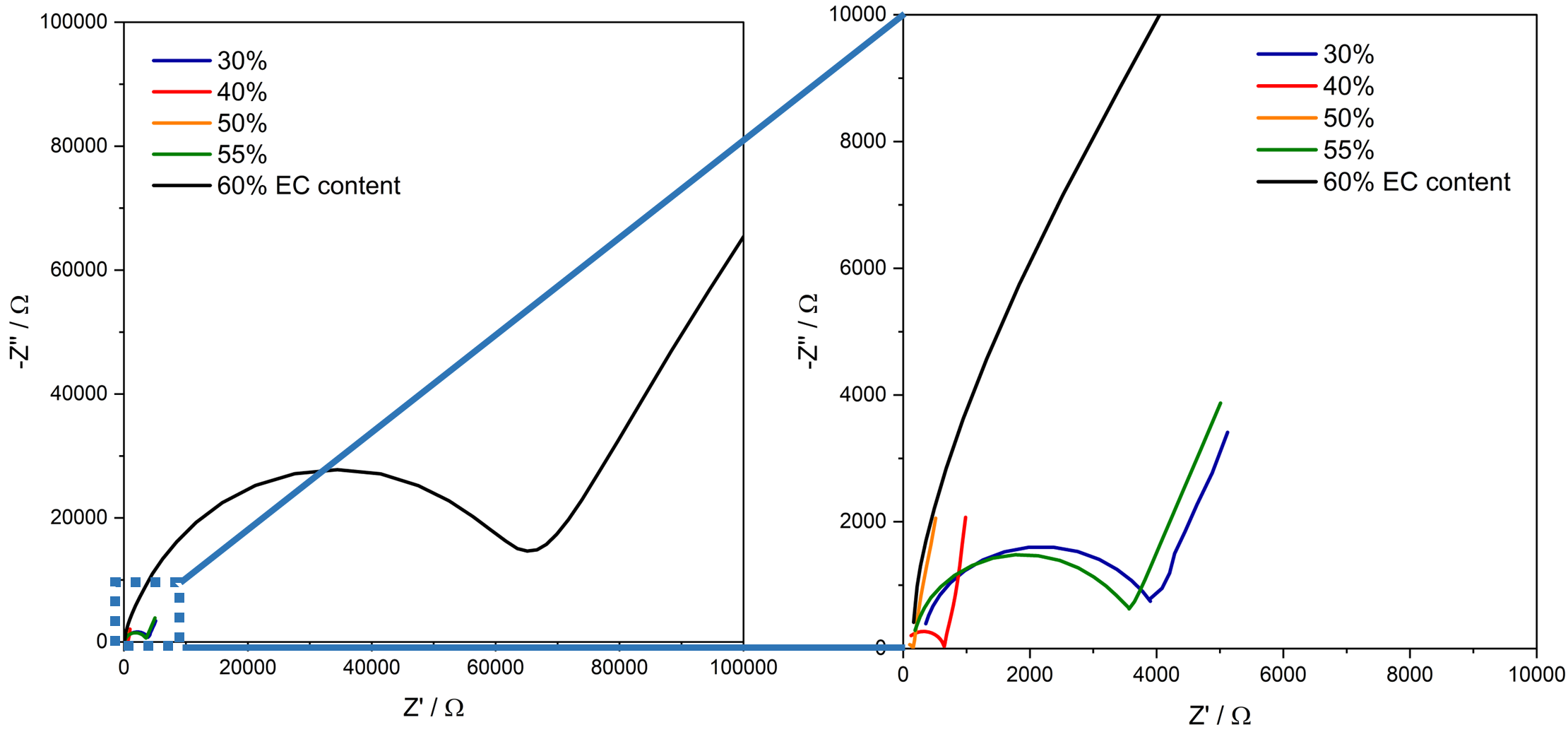


**Figure S6:** Nyquist plots of the EIS data recorded for the determination of the ionic conductivity at 10 °C (as presented in **Figure 3a**) for the SIC-BCEs comprising 30, 40, 50, 55, and 60% EC with a zoom into the low impedance area on the right (as depicted by the dotted frame in the left graph).


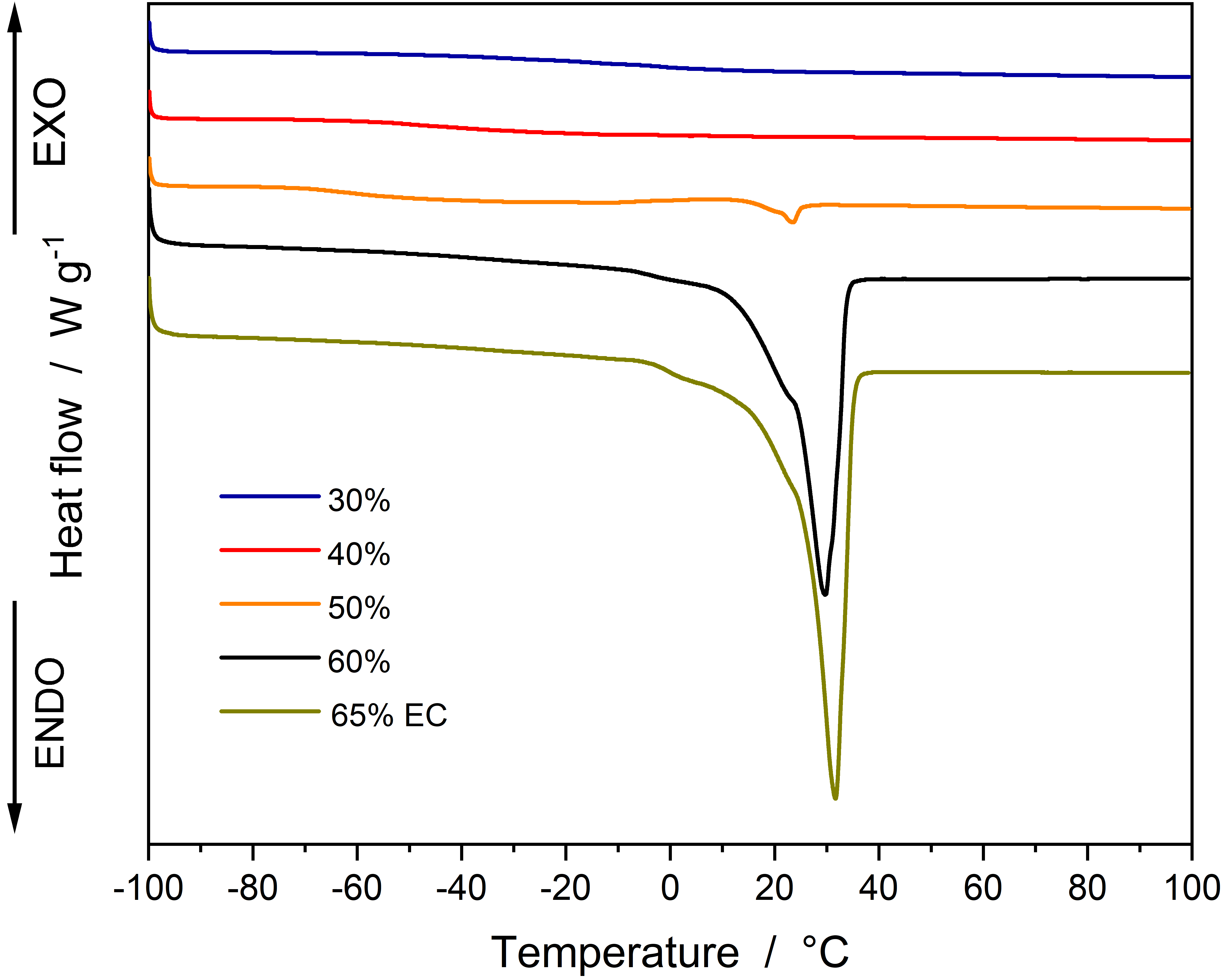


**Figure S7:** DSC curves of the sulfur-doped SIC-BCE comprising various EC contents (30, 40, 50, 60, and 65%).

***
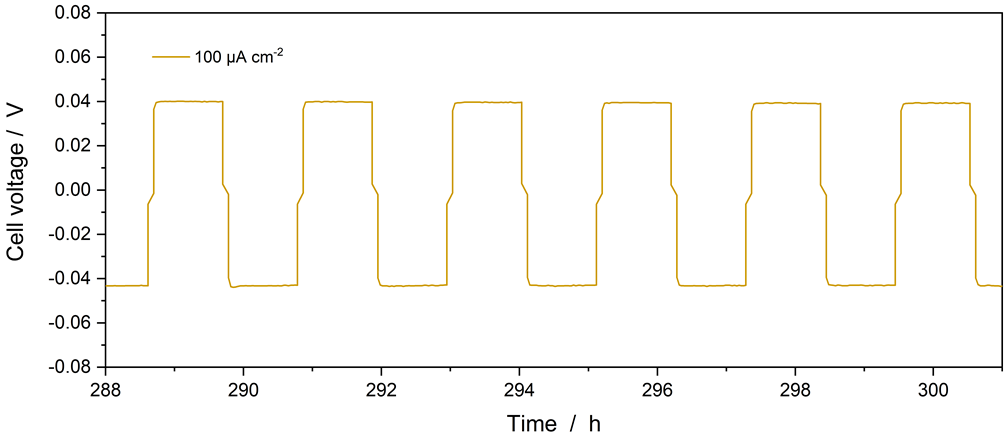
***

**Figure S8:** Zoom into the long-term stripping/plating experiment, displayed in **Figure 3C**.

**
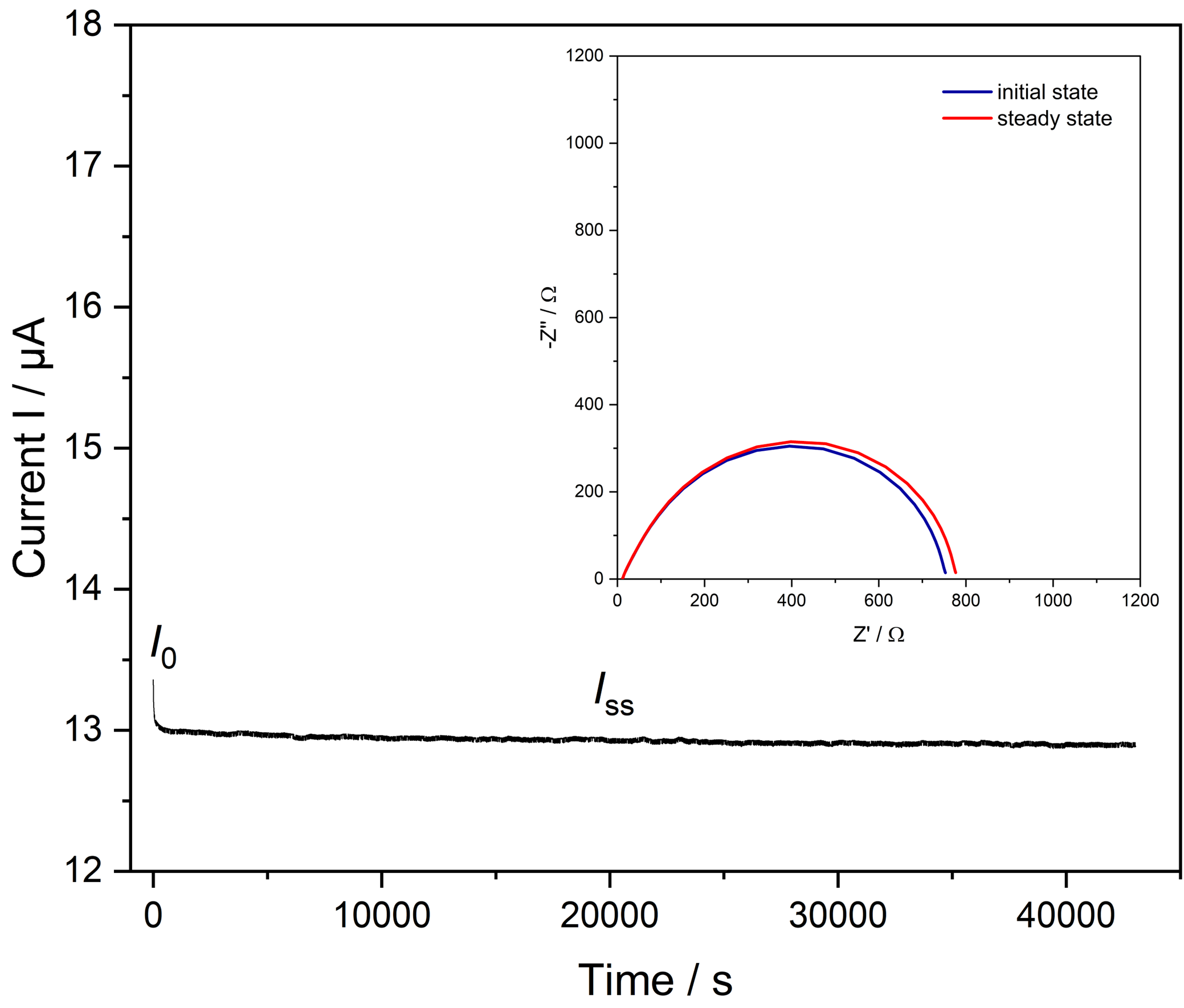
**

**Figure S9:** Current response following the polarization of a symmetric Li||Li cell with 10 mV; inset: EIS spectra recorded before the polarization and at steady state for the determination of the Li^+^ transference number.

**References**

(1) Nguyen, H. D.; Kim, G. T.; Shi, J.; Paillard, E.; Judeinstein, P.; Lyonnard, S.; Bresser, D.; Iojoiu, C. Nanostructured Multi-Block Copolymer Single-Ion Conductors for Safer High-Performance Lithium Batteries. *Energy Environ. Sci.* **2018**, *11* (11), 3298–3309.

(2) Assumma, L.; Iojoiu, C.; Mercier, R.; Lyonnard, S.; Nguyen, H. D.; Planes, E. Synthesis of Partially Fluorinated Poly(Arylene Ether Sulfone) Multiblock Copolymers Bearing Perfluorosulfonic Functions. *J. Polym. Sci. Part A Polym. Chem.* **2015**, *53* (16), 1941–1956.

(3) Assumma, L.; Nguyen, H. D.; Iojoiu, C.; Lyonnard, S.; Mercier, R.; Espuche, E. Effects of Block Length and Membrane Processing Conditions on the Morphology and Properties of Perfluorosulfonated Poly(Arylene Ether Sulfone) Multiblock Copolymer Membranes for PEMFC. *ACS Appl. Mater. Interfaces* **2015**, *7* (25), 13808–13820.
